# Starvation-responsive glycine-rich protein gene in the silkworm *Bombyx mori*

**DOI:** 10.1007/s00360-014-0846-8

**Published:** 2014-08-07

**Authors:** Kiyoko Taniai, Chikara Hirayama, Kazuei Mita, Kiyoshi Asaoka

**Affiliations:** 1 Division of Insect Sciences, National Institute of Agrobiological Sciences, 1-2 Owashi, Tsukuba, Ibaraki 305-8634 Japan; 2 Genetically Modified Organism Research Center, National Institute of Agrobiological Sciences, 1-2 Owashi, Tsukuba, Ibaraki 305-8634 Japan; 3 Agrogenomics Research Center, National Institute of Agrobiological Sciences, 1-2 Owashi, Tsukuba, Ibaraki 305-8634 Japan; 4 Present Address: State Key Laboratory of Silkworm Genome Biology, Southwest University, Tiansheng Road #1, Beibei, Chongqing 400716 China

**Keywords:** *Bombyx mori*, Glycine-rich protein gene, Starvation, Amino acid depletion

## Abstract

Four glycine-rich protein (GRP) genes were identified from expressed sequence tags of the maxillary galea of the silkworm. All four genes were expressed in the maxillary pulp, antenna, labrum, and labium, but none of the genes were expressed in most internal organs. Expression of one of the genes, termed *bmSIGRP*, was further increased approximately fivefold in the mouth region (including the maxilla, antenna, labrum, labium, and mandible) after 24 h of starvation. *bmSIGRP* expression peaked at 24 h and gradually declined during the subsequent 2 days. When a synthetic diet not containing proteins was fed, *bmSIGRP* expression increased significantly in the mouth region to levels similar to that observed in starved larvae. Synthetic diets that lacked vitamins or salts but contained amino acids did not significantly affect *bmSIGRP* expression. These results suggest that amino acid depletion increases *bmSIGRP* expression.

**Electronic supplementary material:**

The online version of this article (doi:10.1007/s00360-014-0846-8) contains supplementary material, which is available to authorized users.

## Introduction

Glycine-rich proteins (GRPs) with quasi-repetitive glycine-rich (GR) motifs (Gly_n_-X_1-2_) have been widely identified in prokaryotes and eukaryotes. These GRPs share similar GR motifs, but their characteristics and functions are diverse. GRPs have been found to act as structural proteins, antimicrobial proteins (Baba et al. 1987; Axén et al. 1997; Park et al. 2000), RNA-binding proteins (Cobianchi et al. 1994; Sato 1994), or stress-inducible proteins. Structural proteins with GR motifs have been identified as components of the body surface, including keratins and loricrins of mammalian skin (Mehrel et al. 1990), chorion and egg-shell proteins of invertebrates (Hamodrakas et al. 1985; Sudo et al. 1997), insect cuticular proteins (Højrup et al. 1986; Charles et al. 1992; Andersen et al. 1995), cement proteins of ticks (Maruyama et al. 2010), and plant cell-wall proteins (Keller 1993). In the silkworm *Bombyx mori*, the expression of several *GRP* genes predicted to encode cuticular proteins was identified in epidermal cells (Suzuki et al. 2002; Zhong et al. 2006). Stress-inducible GRPs are extensively documented in plants. Plant GRPs encompass five classes based on their primary structures (Mousavi and Hotta 2005; Mangeon et al. 2010). Each class contains several GRPs that are transcriptionally regulated by different types of stresses and factors, such as water stress, wounding, infection, cold shock, abscisic acid treatment, and salicylic acid treatment (Sachetto-Martins et al. 2000). Three cuticular *GRP* genes have been identified in an insecticide-resistant strain of the Colorado potato beetle *Leptinotarsa decemlineata* (Say); all are strongly induced by insecticides (Zhang et al. 2008).

In this study, we identified four *GRP* genes from expressed sequence tags (ESTs) of maxillary galea from silkworm, and found that one is strongly induced by starvation and amino acid depletion. To the best of our knowledge, this is the first GRP gene to be identified as being inducible by nutritional stress in insect.

## Materials and methods

### Identification and characterization of *GRP* genes

The EST library of the maxillary galea (fmxg-EST) of 5th-instar *B. mori* larvae was previously described (Yoshizawa et al. 2011). The GRP genes were manually identified in the library. The signal peptide was predicated using the SignalP 4.1 program (http://www.cbs.dtu.dk/services/SignalP/), and the isoelectric point and molecular size were predicated using the GENETYX-MAC software (version 14; GENETYX Co, Tokyo). The genome location of the gene was determined through a scaffold sequence search of the KAIKObase database (http://kaikoblast.dna.affrc.go.jp).

### Insects and diet

The *B. mori* strains N137 × C146 and N601 × C601 were reared at 25 °C under a photoperiod of 12 h light/12 h dark and fed the Silkmate 2S and Silkmate L4-M artificial diets, respectively. The Silkmate 2S diet contains a high amount of mulberry leaves, whereas the Silkmate L4-M diet contains only a few percent mulberry leaves (the details of the recipe are not indicated by the manufacturer, Nippon Nosan Co. Ltd.). The N601 × C601 strain is relatively polyphagous and eats the L4-M diet. For nutrition deprivation analyses, the N601 × C601 larvae were transferred from the L4-M diet to a synthetic diet (Supplemental Table 1) established by Yanagawa et al. (1995) after the fourth molt. The synthetic diet, designated SAD100 (Synthetic Artificial Diet 100 %), contains all of the nutrients necessary for larval growth. To prepare the diets lacking amino acids (SAD-AA), inorganic salts (SAD-IS), and vitamins (SAD-V), an equivalent amount (weight) of cellulose powder was added in place of the deficient nutrients.

### Stress test

Five 1-day-old fifth-instar larvae were placed in a plastic cup (12 cm in diameter) with the Silkmate 2S diet. The cup was then covered with a lid and placed at 25 °C and 60–65 % humidity; these conditions were considered the control conditions. To test starvation, larvae were placed in an empty cup with a lid. To test low- and high-humidity conditions, larvae in a cup (without a lid) were placed at 25 °C in a desiccator chamber, which was adjusted to 11 % or 84 % humidity using a saturated LiCl or KCl solution, respectively. The humidity was confirmed using a hygrometer. After 24 h of treatment, the larval mouth region containing all of the oral appendages (antennae, maxilla, labrum, labium, and mandible) was removed and analyzed by quantitative reverse transcription-polymerase chain reaction (qRT-PCR).

### qRT-PCR

Tissues were removed from 1- to 2-day-old fifth-instar larvae, immediately homogenized in 1 ml of cold ISOGEN reagent (Nippon Gene) using a polypropylene pestle, and maintained at −80 °C until use. Total RNA was separated from the DNA, proteins, and other substances according to the manufacturer’s instructions. cDNA was synthesized from 1 μg of total RNA using a Transcriptor First-Strand cDNA Synthesis Kit (Roche) with an anchored oligo(dT)_18_ primer. qRT-PCR analysis was performed in a 20-µl reaction volume containing 0.5 µM primers, 1× LightCycler 480 SYBR Green I Master Mix, and 4 µl of the template sample or a standard plasmid. The standard plasmids were prepared by amplifying the coding regions of the *GRP* genes by PCR and inserting them into the pGEM-T Easy plasmid (Promega). Standard curves were generated using the signals from serial dilutions (0.1–1,000 pg) of the plasmids, and the crossing points of the standards and the samples were used to determine the amount of transcript in each sample. PCR was conducted using the following temperature program in a Light Cycler 480 Real-Time PCR System (Roche): 95 °C for 5 min followed by 45 cycles at 95 °C for 5 s, 58 °C for 5 s, and 72 °C for 10 s. Following the PCR, the absence of unwanted by-products was confirmed through melting curve analysis. The amount of transcripts was normalized to the amount of the actin *A3* gene (Mounier and Prudhomme 1986) in each sample, which was analyzed using the TaqMan system as described previously (Yoshizawa et al. 2011). The primers used in the experiments are listed in Supplemental Table 2.

### Quantification of amino acids in hemolymph

The concentration of alpha-amino groups in the hemolymph was measured through the Ninhydrin reaction (Yemm and Cocking 1954). The hemolymph was collected by cutting the abdominal legs on ice and removing the hemocytes in the hemolymph by centrifugation at 3,000×*g* for 10 min. The proteins in the supernatant were precipitated through the addition of four volumes of 10 % trichloroacetic acid (TCA), incubating on ice for 15 min, and centrifuging at 14,000 × *g* for 10 min. To remove the TCA, the supernatant was extracted with an equal volume of diethyl ether. The hemolymph samples were maintained at −30 °C until use. The samples were then mixed with 0.2 M citric acid (pH 5.0, at a volumetric ratio of 1:9) and then with an equal volume of the Ninhydrin test solution (Wako). The mixture was then boiled for 15 min and immediately cooled on ice. The solution was mixed with five volumes of 50 % ethanol, and the absorbance at 570 nm was measured. A standard curve was prepared using varying concentrations of an arginine solution (0.1, 0.5, 1, and 5 mM). The experiments were performed three times, and the significance of differences between SAD-AA and SAD100, and between SAD-AA and starvation, was estimated by the Student’s *t* test with *P* < 0.01 accepted as significant.

## Results

### Identification of *GRP* genes

**Fig. 1:**
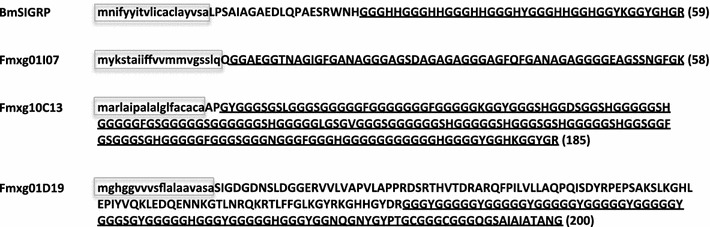
Deduced amino acid sequences of the four *GRP* genes. The *small letters* indicated in *boxes* correspond to the predicted signal peptides. *Underlined letters* depict GR motifs. *Numbers in parentheses* indicate the number of amino acids in the predicted mature proteins

We identified four *GRP* genes, *bmSIGRP*, *fmxg10C13*, *fmxg01I07*, and *fmxg01D19*, in the fmxg-EST library, which was constructed using the N137 × C146 strain. The frequencies of the four *GRP* genes in the fmxg-ESTs were 173 (4.0 %), 73 (1.7 %), 48 (1.0 %), and 32 (0.7 %), respectively, in a total of 4,267 reads, indicating that each is highly expressed in the maxillary galea. Two of the genes, *bmSIGRP* and *fmxg01D19*, were previously annotated as cuticular *GRP* genes (*cpg36* and *cpg10*, respectively) (Futahashi et al. 2008), but evidence was not provided that these genes are cuticular protein genes. The other two genes, *fmxg10C13* and *fmxg01I07*, were identified *de novo* in this study. All four *GRP* genes encode GRPs with a typical GR motif of a (Gly_2-7_-X_1-2_) repeat sequence in the glycine-rich regions and contain a predicted signal peptide (Fig. 1). The characteristics of the deduced mature proteins, including molecular size, p*I*, and glycine content, vary (Table 1). The sequences of the deduced BmSIGRP and Fmxg01D19 proteins lack the GR motif at the N terminus, whereas those of Fmxg01I07 and Fmxg10C13 contain the GR motif throughout the protein. In particular, the glycine content of Fmxg10C13 is extremely high (71.4 %). The predicted p*I* of three of the GRPs is basic, whereas that of Fmxg01I07 is acidic (3.87). These differences in the p*I* value are due to the different X residues in the GR motifs: the X residues in BmSIGRP are mostly His, the X residues in Fmxg01I07 vary (Ala, Phe, Asn, Ser, and Glu), the X residues in Fmxg10C13 are mostly Phe and His, and the X residues in Fmxg01D19 are mostly Tyr and His. The locations of the four *GRP* genes in the genome also differ (Table 1). *bmSIGRP* was mapped to chromosome 19, *fmxg01D19* and *fmxg10C13* were mapped to chromosome 5, and *fmxg01I07* was mapped to chromosome 16. These quite different characteristics suggest that the four GRPs have different roles.

**Table 1:** Characteristics of the deduced mature protein of four GRP genes

Protein name	MW (kDa)	p*I*	Gly (%)	Ch	Hydrophobic residues (%)	Acc. No.
BmSIGRP	5.8	8.21	40.7	19	57.63	AB839176
Fmxg10C13	13.7	9.77	71.4	5	75.86	AB839178
Fmxg01I07	4.7	3.87	44.8	16	77.30	AB839177
Fmxg01D19	19.4	9.14	35.6	5	58.79	AB839179

*MW* molecular weight, *pI* isoelectric point, *Ch* chromosome

### Distribution of the four *GRP* genes

**Fig. 2:**
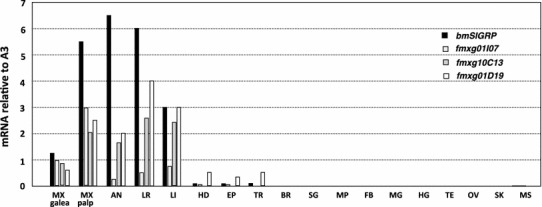
Distribution of the expression of the four *GRP* genes. Organs were removed from 5–10 larvae on days 1–2 of the fifth instar, and levels of the transcript corresponding to each of the four *GRP* genes were quantified by qRT-PCR. The *data* represent the means of independent experiments (*n* = 2–5). The expression of the actin *A3* gene was used as an internal control. *MX* maxilla, *AN* antenna, *LR* labrum, *LI* labium, *HD* head without the oral appendages and eyes, *EP* epidermis of the body, *TR* trachea, *BR* brain, *SG* subesophageal ganglion, *MP* malpighian tube, *FB* fat body, *MG* midgut, *HG* hindgut, *TE* testis, *OV* ovary, *SK* silk gland, *MS* muscle

The expression of the four *GRP* genes was investigated in the oral appendages of fifth-instar larvae of the N137 × C146 strain: the maxillary galea, maxillary pulp, antenna, labrum and labium, the head lacking all of the previously listed oral appendages, epidermis, trachea, and various internal organs (Fig. 2). All of the four genes were found to be highly expressed in all of the oral appendages. Three of the genes, *bmSIGRP*, *fmxg01I07*, and *fmxg01D19*, were weakly expressed in the head, trachea, and epidermis. However, none of the four genes was expressed in the other internal organs, such as the Malpighian tube, fat body, midgut, hindgut, testis, ovary, silk gland, or muscle.

### Starvation increases *bmSIGRP* expression

**Fig. 3:**
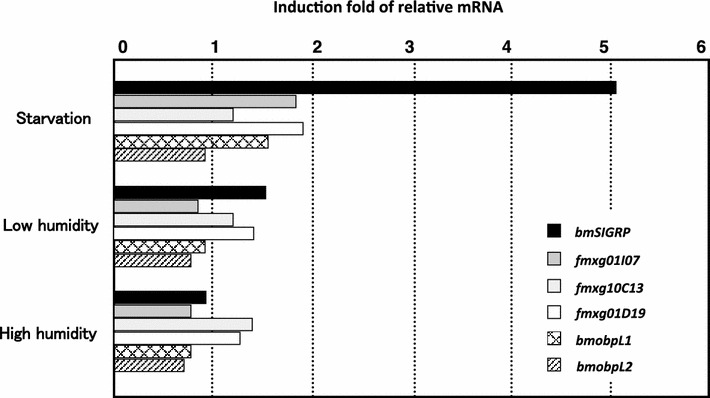
Starvation increases *bmSIGRP* expression. After the fourth ecdysis, the larvae were starved or placed under low humidity (11 %) or high humidity (84 %) conditions for 24 h, and the expression of *bmSIGRP* in the mouth region was analyzed by qRT-PCR. The expression was normalized using the actin *A3* gene as an internal control. Induction folds were calculated based on the expression level in control larvae that were reared on L4-M diet at 25 °C and 60–65 % humidity. Ten larvae were used for each experiment; *bars* represent the mean values of two experiments. *bmobpL1* and *bmobpL2* are odorant-binding protein genes that were used as negative controls

**Fig. 4:**
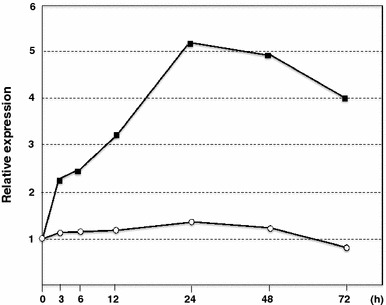
Expression time course of *bmSIGRP.* After the fourth ecdysis, larvae were fed an artificial diet for 18 h and then fed or starved for 3 days. The mouth region was collected from three larvae at the indicated time points, and the *bmSIGRP* transcript was quantified by qRT-PCR. Expression of the actin *A3* gene was used as an internal control

We used the N137 × C146 strain to examine whether the four *GRP* genes are responsive to stress because many plant *GRP* genes are regulated by stress. Immediately after the fourth molt, larvae were starved or exposed to low or high humidity for 24 h, and then the mouth region was analyzed for *GRP* gene expression. Two odorant-binding protein genes (*bmobpL1* and *bmobpL2*) that are stably expressed in the mouth region (Yoshizawa et al. 2011) were used as negative controls. Three *GRP* genes, *fmxg10C13*, *fmxg01I07*, and *fmxg01D19*, were not significantly changed by any of these stresses, similar to the control genes. In contrast, the expression of *bmSIGRP* increased approximately fivefold in response to starvation (Fig. 3). Neither high nor low humidity affected *bmSIGRP* expression. Thus, we named the gene *bmSIGRP* (*Bombyx mori* Starvation Inducible Glycine-Rich Protein gene). We next analyzed an expression time course of *bmSIGRP* after starvation. To ensure that the start time of the starvation was the same relative to the larval stage under all conditions, the larvae were fed for 18 h after the fourth molt, after which either starvation was begun or feeding was continued for 3 days. The mouth region was collected from three to five larvae at each time point, at 3, 6, 12, 24, 48, and 72 h after the start of starvation. The PCR results revealed that the expression of *bmSIGRP* increased rapidly, reached a maximum at 24 h, and then declined gradually in the subsequent 2 days (Fig. 4).

### Amino acid depletion increases bmSIGRP expression

**Fig. 5:**
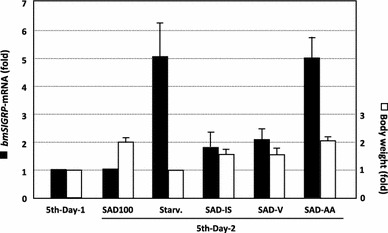
Amino acid depletion increases *bmSIGRP* expression. After the fourth ecdysis, larvae were fed experimental diets (SAD100, SAD-IS, SAD-V, or SAD-AA) or starved (Starv.) for 24 h. The *bmSIGRP* transcript was quantified by qRT-PCR. Expression of the actin *A3* gene was used as an internal control. *Black bars* represent the relative levels of *bmSIGRP* mRNA in the mouth region induced after each treatment. *White bars* indicate the fold increase in body weight. *Error bars* indicate the standard deviations for three independent experiments

To determine the factor(s) involved in the increased *bmSIGRP* expression induced by starvation, we prepared several synthetic diets lacking a particular nutritional component. The complete synthetic diet contained no proteins, but instead contained 18 amino acids. None of the larvae, even those of the relatively polyphagous *B. mori* strain (N601 × C601), ate the diet that lacked sucrose. Therefore, we could not examine the effect of sugar depletion on *bmSIGRP* expression. In contrast, the larvae ate the diet lacking amino acids (SAD-AA), and their body weights increased equivalently to the larvae that ate the normal artificial diet (L4-M) or the complete synthetic diet (SAD100) (Fig. 5). The larvae also ate the diet lacking inorganic salts (SAD-IS) and the diet lacking vitamins (SAD-V), although they consumed less than with the L4-M and SAD-100 diets. After 24 h of diet consumption, *bmSIGRP* expression was significantly increased in the mouth region of larvae that ate the SAD-AA diet, equivalent to the *bmSIGRP* expression increase observed in starved larvae. The other diets did not significantly affect *bmSIGRP* expression. Thus, amino acid depletion increases the expression of *bmSIGRP*.

**Fig. 6:**
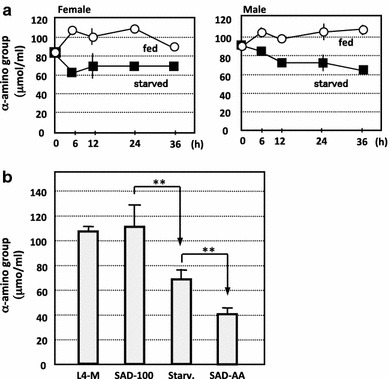
Amino acid concentration in larval hemolymph. **a** Concentration of alpha-amino groups in the hemolymph determined by the Ninhydrin reaction. Hemolymph was collected from larvae at 0, 6, 12, 24, and 36 h after initiation of feeding of the L4-M diet (*white circles*) or of starvation (*black squares*) immediately after the fourth ecdysis. *Vertical bars* on the symbols indicate the standard error from three independent experiments, and *symbols* *without bars* indicate that the standard error is too small to be depicted in the figure. **b** Concentration of alpha-amino groups in the hemolymph of larvae (mix of female and male) at 24 h after the fourth ecdysis. Standard errors were calculated from three independent experiments. The *double stars* between SAD100 and Starv. (starvation), and between Starv. and SAD-AA, indicate significant differences (*P* > 0.01) as determined through a *t* test of data from three independent experiments

We then analyzed the effect of starvation on the amino acid concentration in the larval hemolymph. Hemolymph was collected at 0, 6, 12, 24, and 36 h after the fourth ecdysis from starved N601 × C601 larvae or N601 × C601 larvae fed L4-M, and the concentration of alpha-amino groups was determined using the Ninhydrin reaction. The total alpha-amino group concentration in the hemolymph was higher at 12 h after the start of feeding, and the level was maintained for the next 24 h. In contrast, the total alpha-amino group concentration had decreased in hemolymph of the starved larvae, both female and male, at 12 h after the fourth ecdysis, and the level remained low during the next 24 h (Fig. 6a). These results indicate that starvation affects the amino acid concentrations in the hemolymph. We next compared the amino acid concentration in the hemolymph of both male and female larvae that were fed SAD100, SAD-AA, or L4-M, or starved for 24 h after the fourth ecdysis. The amino acid concentration in larvae that were fed SAD100 was equivalent to that in larvae that were fed L4-M, but significantly lower (*P* < 0.01) in larvae that were fed SAD-AA (Fig. 6b). The concentration in larvae that were fed SAD-AA was even lower than that in starved larvae. These results indicate that both starvation and amino acid depletion result in a decrease in the amino acid concentration in the hemolymph.

## Discussion

Several stress-responsive GRPs with the GR motif have been identified, mainly in plants (Sachetto-Martins et al. 2000). A few beetle cuticular *GRP* genes that are induced by an insecticide are thought to provide strength to the beetle’s cuticle and therefore help it survive under severe environmental conditions (Zhang et al. 2008). We identified four *GRP* genes that are highly expressed in oral appendages of the silkworm (Fig. 2). This expression pattern suggests that these GRPs are related to feeding behavior or to the complex structure of the oral appendages. Although the distribution of the expression of these four *GRP* genes is similar, the functions of the genes may be different because the deduced proteins differ in characteristics such as molecular size, p*I*, and amino acid composition. We examined whether some environmental stresses regulate the four *GRP* genes and found that one of the genes, *bmSIGRP*, shows significantly increased expression after starvation (Fig. 3). In contrast, the expression of the other three GRPs was not altered by starvation. Many *GRP* genes have been reported to be upregulated by dryness (Sachetto-Martins et al. 2000). However, environmental humidity stress did not affect *bmSIGRP* expression, suggesting that water depletion caused by starvation is unrelated to *bmSIGRP* regulation. We next examined the effects of nutritional depletion stress on *bmSIGRP* expression using synthetic diets. If larvae are reared on a synthetic diet beginning as neonates, their growth speed becomes slow and body sizes become diverse. Therefore, a normal artificial diet was used to grow the larvae until the end of the fourth instar. The larvae were then transferred to the synthetic diet after the fourth ecdysis to test nutritional stress. The possibility exists that simply a change in diet causes nutritional stress in the larvae. However, this possible stress did not affect *bmSIGRP* expression because the *bmSIGRP* transcript levels were equivalent in larvae that were reared continuously on the L4-M diet and those that were reared on the SAD100 diet for 24 h. Using several different synthetic diets that lack a particular nutrient, we found that amino acid depletion strongly induced *bmSIGRP* expression to a level similar to that of starvation (Fig. 5). Thus, *bmSIGRP* is regulated by amino acid availability.

Regulation of *bmSIGRP* expression might be mediated by the taste organs in the mouth region, the amino acid concentration in the hemolymph, or the amino acid concentration in cells. Of these possibilities, we examined whether the amino acid concentration in the hemolymph was related to *bmSIGRP* expression. Silkworms require ten essential amino acids for normal growth, and deprivation of one of these amino acids rapidly decreases their total protein synthesis (Horie and Inokuchi 1978). If protein synthesis is ceased, the amino acid concentration in the hemolymph may not change. To clarify this issue, we analyzed the concentration of amino acids in the hemolymph during starvation or amino acid depletion. The amino acid concentration decreased in both larvae that were starved and those that were depleted of amino acids after 24 h (Fig. 6a). Notably, the concentration was significantly lower (*P* < 0.01) in larvae that were fed SAD-AA than in starved larvae (Fig. 6b), most likely because the larvae that were fed the SAD-AA diet used energy for the feeding activity, resulting in a higher rate of consumption of the amino acids in the hemolymph in comparison to the starved larvae. These results suggest that the amino acid concentration in the hemolymph is related to the mechanism of *bmSIGRP* gene regulation.

Depletion of amino acids downregulates the target of the rapamycin (TOR) signaling pathway (Wullschleger et al. 2006), which is widely conserved from yeast to mammals (Colombani et al. 2003; Goberdhan et al. 2005). Because a *TOR* gene has been isolated from *B. mori* (Zhou et al. 2010), determining whether the regulation mechanism of *bmSIGRP* is related to regulation mechanism of the TOR signaling pathway may be possible. In addition, identifying the function of *bmSIGRP* is important. Predicting the function of BmSIGRP based on those of other similar proteins is difficult because GRPs have very diverse functions (Mangeon et al. 2010). To elucidate the function of *bmSIGRP*, attempts to overexpress *bmSIGRP* in baculovirus will be useful, and knocking out *bmSIGRP* using a TALEN system, a recently established genetic tool in insects (Ma et al. 2012; Takasu et al. 2013), will be interesting.

## Electronic supplementary material

Below is the link to the electronic supplementary material.

Supplementary material 1 (DOC 78 kb)

Supplementary material 2 (DOC 35 kb)
