# Supplementary material for: Starvation-responsive glycine-rich protein gene in the silkworm Bombyx mori
Source: J Comp Physiol B. 2014 Aug 7;184(7):827–34. doi: 10.1007/s00360-014-0846-8 (PMC4171585; doi:10.1007/s00360-014-0846-8)
Supplement: Supplementary file 1 — Supplementary material 1 (DOC 78 kb) [file 360_2014_846_MOESM1_ESM.doc]

Supplemental Table 1. Composition of SAD100

| Substance | |  |
| --- | --- | --- |
| Amino acids | |  |
|  | Arginine-HCl | 1.27 g |
|  | Histidine | 0.6 g |
|  | Isoleucine | 0.95 g |
|  | Leucine | 1.85 g |
|  | Lysine-HCl | 2.13 g |
|  | Methionine | 0.27 g |
|  | Phenylalamine | 1.16 g |
|  | Threonine | 1.05 g |
|  | Tryptophan | 0.32 g |
|  | Valine | 1.25 g |
|  | Prolone | 1.16 g |
|  | Alanine | 1.37 g |
|  | Glycine | 1.22 g |
|  | Serine | 1.11 g |
|  | Tyrosine | 0.76 g |
|  | Cystine | 0.16 g |
|  | Glutamate-K | 3.62 g |
|  | Aspartate-K | 3.68 g |
| Inorganic salts | |  |
|  | K_2_HPO_4_ | 2.25 g |
|  | KCl | 0.5 g |
|  | CaCO_3_ | 1.0 g |
|  | MgSO_4_ | 0.3 g |
|  | ZnCl | 5 mg |
|  | FePO_4_ | 0.1 g |
| Vitamines | |  |
|  | Biotin | 0.2 mg |
|  | Choline chloride | 150 mg |
|  | Foric acid | 0.2 mg |
|  | Inositol | 0.2 g |
|  | Nicitinic acid | 10 mg |
|  | Ca-Pantothenate | 15 mg |
|  | Pyridoxine-HCl | 3 mg |
|  | Riboflavin | 2 mg |
|  | Thiamine | 2 mg |
|  | Ascorbic acid | 2 g |
| Citric acid | | 4 g |
| Sucrose | | 10 g |
| Starch, Potato | | 10 g |
| Soybean oil, refined | | 10 ml |
| Morin | | 0.5 g |
| Chlorogenic acid | | 0.2 g |
| β-Sitosterol | | 0.3 g |
| Cellulose powder | | 33.915 g |
| Agar | | 8 g |
| Proponic acid | | 10 ml |
| Water | | 250 ml |
