# Supplementary material for: Starvation-responsive glycine-rich protein gene in the silkworm Bombyx mori
Source: J Comp Physiol B. 2014 Aug 7;184(7):827–34. doi: 10.1007/s00360-014-0846-8 (PMC4171585; doi:10.1007/s00360-014-0846-8)
Supplement: Supplementary file 2 — Supplementary material 2 (DOC 35 kb) [file 360_2014_846_MOESM2_ESM.doc]

**Supplemental Table 2. Primers used for qRT-PCR.**

|  | Forward | Reverse |
| --- | --- | --- |
| *bmSIGRP* | 5’-CTCGCATATGTCTCCGCTCT-3’ | 5’-GCCATGATTCCAACGAGACT-3’ |
| *fmxg10C13* | 5’-GGTGGATTTGGAGGAGGAAG-3’ | 5’-CCACCTTTGTGACCACCATA-3’ |
| *fmxg01I07* | 5’-GCCGAAGGAGGAACTAATGC-3’ | 5’-ATTAGCGCCGAATTGGAAAC-3’ |
| *fmxg01D19* | 5’-GAGGCGAGCGTGTAGTTCTC-3’ | 5’-CTGAGGGTTCAGGACGGTAG-3’ |
